# “Miswak” Based Green Synthesis of Silver Nanoparticles: Evaluation and Comparison of Their Microbicidal Activities with the Chemical Synthesis

**DOI:** 10.3390/molecules21111478

**Published:** 2016-11-06

**Authors:** Mohammed Rafi Shaik, Ghadeer H. Albalawi, Shams Tabrez Khan, Merajuddin Khan, Syed Farooq Adil, Mufsir Kuniyil, Abdulrahman Al-Warthan, Mohammed Rafiq H. Siddiqui, Hamad Z. Alkhathlan, Mujeeb Khan

**Affiliations:** 1 Department of Chemistry, College of Science, King Saud University, P.O. Box 2455, Riyadh 11451, Saudi Arabia; rafiskm@gmail.com (M.R.S.); ghadeer8830@hotmail.com (G.H.A.); mkhan3@ksu.edu.sa (M.K.); sfadil@ksu.edu.sa (S.F.A.); mufsir@gmail.com (M.K.); awarthan@ksu.edu.sa (A.A.-W.); rafiqs@ksu.edu.sa (M.R.H.S.); 2 Faculty of Science, University of Tabuk, P.O. Box 741, Tabuk 71491, Saudi Arabia; 3 Department of Zoology, College of Science, King Saud University, P.O. Box 2455, Riyadh 11451, Saudi Arabia; stkhan@ksu.edu.sa

**Keywords:** silver nanoparticles, natural products, green chemistry, anti-microbial activity

## Abstract

Microbicidal potential of silver nanoparticles (Ag-NPs) can be drastically improved by improving their solubility or wettability in the aqueous medium. In the present study, we report the synthesis of both green and chemical synthesis of Ag-NPs, and evaluate the effect of the dispersion qualities of as-prepared Ag-NPs from both methods on their antimicrobial activities. The green synthesis of Ag-NPs is carried out by using an aqueous solution of readily available *Salvadora persica* L. root extract (RE) as a bioreductant. The formation of highly crystalline Ag-NPs was established by various analytical and microscopic techniques. The rich phenolic contents of *S. persica* L. RE (Miswak) not only promoted the reduction and formation of NPs but they also facilitated the stabilization of the Ag-NPs, which was established by Fourier transform infrared spectroscopy (FT-IR) analysis. Furthermore, the influence of the volume of the RE on the size and the dispersion qualities of the NPs was also evaluated. It was revealed that with increasing the volume of RE the size of the NPs was deteriorated, whereas at lower concentrations of RE smaller size and less aggregated NPs were obtained. During this study, the antimicrobial activities of both chemically and green synthesized Ag-NPs, along with the aqueous RE of *S. persica* L., were evaluated against various microorganisms. It was observed that the green synthesized Ag-NPs exhibit comparable or slightly higher antibacterial activities than the chemically obtained Ag-NPs.

## 1. Introduction

The development of various green procedures for the preparation of metallic nanoparticles (NPs) is highly desirable in the current scenario, because of rising environmental apprehensions caused by the climate change [1,2]. Usually, various chemical and physical methods have been employed to prepare different metallic NPs; however, these methods are costlier and less environmentally friendly. Moreover, the organic waste generated during these methods greatly increases the environmental pollution. Therefore, there is a growing need to develop alternate processes for the synthesis of metallic NPs that are cost effective, eco-friendly, and more sustainable.

In this context, the strategies of green chemistry have considerable contributions to the synthesis of metallic NPs [3]. Following these green chemistry strategies, various chemical syntheses of NPs can be conveniently transformed into greener procedures by the appropriate choice of solvents, reductants, and stabilizers [4]. So far, numerous green methods have been used for the synthesis of various metallic NPs under different physiological and eco-friendly conditions [5]. These methods include electrochemical, microwave, sonochemical, supercritical liquids, ionic liquids, etc. [6,7,8]. Recently, the trend of employing natural materials, like, marine organisms, microorganisms, proteins, and plant extracts (PE) in the green preparation of metallic NPs has gained enormous popularity in the scientific community [9,10]. Particularly, the green preparation of metallic NPs using PE, has efficiently accomplished various principles of green chemistry, as it is less expensive, eco-friendly, and greatly reduces the environmental hazards by reducing the usage of toxic chemicals and producing less waste [11].

Among, various metallic NPs, silver nanoparticles (Ag-NPs) have drawn considerable attention of researchers and scientists because of their wide range of applications in the progress of new technologies in various fields, including, material sciences, nanoscience, electronics, and medicine [12,13,14]. Ag-NPs have been widely applied in different fields, including, as an anti-microbial agent in various applied materials, as effective coating agents, efficient biosensors, and optical receptors, as well as catalysts in different chemical reactions. Additionally, Ag-NPs have also been found to possess several biological properties—anti-HIV [15], larvicidal [16], and anti-platelets [17], etc. Moreover, Ag-NPs have also been used in textiles [18], water desalination [19], and food packaging [20], etc.

Most of the properties possessed by Ag-NPs are largely based on their morphology and size of the NPs. It is extensively reported in various studies that the spherical shaped silver NPs with small size are more effective compared to the other morphologies of NPs [21,22,23]. Recently, various reports concerning the controlled synthesis of Ag-NPs by different methods have been described. However, little attention has been paid so far to control the morphology and size of the Ag-NPs using PE as reductants [24,25,26]. Comparatively, chemical synthesis shows better control on monodispersity, size distribution, and enhanced dispersibility by the use of additional stabilizers [12,27]. Morphology, size, and size distribution of NPs are important parameters which define the biological fate, toxicity, and specific targeting ability of Ag-NPs. Therefore, controlling these parameters is very important during the PE mediated synthesis of Ag-NPs, in order to achieve maximum advantages in their biological applications. Particularly, significant work needs to be done towards the enhancement of the dispersibility of Ag-NPs to further improve their antimicrobial properties [28,29].

Natural products obtained from different organisms including bacteria, fungi, and plants are proving to be valuable reducing agents for the synthesis of NPs as they exhibit significant chemical diversity yielding wide spectrum of biological activities [30]. These natural products have tremendous varieties in their chemical structures and hold noteworthy pharmacological properties, which have been effectively utilized for various applications, including pharmaceuticals [31]. The pharmacologically active phytoconstituents present in the PE not only expedite the preparation of the Ag-NPs by performing as effective reductants, but also stabilize the surface of NPs [21]. In addition, the application of phytoconstituents as an in situ stabilizing ingredient accelerates the synthesis to be performed under simple conditions [32]. Moreover, it is significant to point out that the methods of PE mediated synthesis of Ag-NPs, which fulfil many of the basic rules of green chemistry, are relatively more biocompatible compared to the chemical methods, and hence these methods can be potentially exploited in large-scale biological applications [24].

Furthermore, green synthesis may improve the bioactivity of the NPs through the following possible routes. Firstly, it may result in the capping of NPs with biomaterials enhancing their colloidal stability and consequently enhancing their bioavailability [33,34]. Thus, the increased bioavailability results in improved bioactivity as demonstrated by Khan et al. [35]. Similarly, in a recent study cyclodextrin derivative was used as capping/reducing agent during the preparation of Ag-NPs [36]. The binding of cyclodextrin to the Ag-NPs resulted in an enhancement of the bioactivity of this supramolecular system, which was used as a drug carrier. Furthermore, the enhanced interactions between the Ag**amCD** (poly-{6-[3-(2-(3-aminopropylamino)ethylamino) propylamino]}-(6-deoxy)-β-cyclodextrin) system and antibiotics, due to the excellent binding ability of cyclodextrin further resulted in a synergistic improvement of the antibacterial activity of the system. Moreover, the capping of NPs with natural compounds may also facilitate the binding of the synthesized NPs to different bio-surfaces and biomolecules, and may also help NPs cross certain bio membranes [37]. In fact, this may impart NPs with a very valuable pharmaceutical property of selective binding to specific biomolecules or surfaces. Moreover, many natural compounds may have their own bioactivity, which may supplement the bioactivity of the NPs synthesized using green approach. Hence, NPs synthesized through a green approach show improved bioactivity compared to the NPs synthesized through chemical synthesis [38].

**Figure 1:**
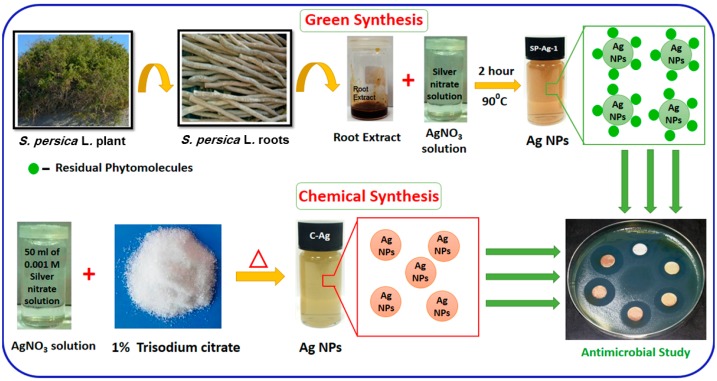
Graphical representation of green and chemical syntheses of silver nanoparticles (Ag-NPs) using *S. persica* L. RE and trisodium citrate, respectively, and evaluation of their microbicidal activities against various bacterial strains.

In the present work, we have used *Salvadora persica* L. root extract (RE) as a bioreducing agent for the green synthesis of Ag-NPs. *S. persica* L. is commonly known as “Miswak” and has rich contents of alkaloids, flavonoids, terpenoids, saponins, and tannins, which makes it attractive for the green synthesis of various materials including graphene [39]. In order to further exploit the potential of *S. persica* L. RE towards the green synthesis of metallic NPs, herein we report the preparation of Ag-NPs using this RE both as a reducing and stabilizing agent. Furthermore, during this study, various potential properties of the as-prepared green synthesized Ag-NPs, including their dispersibility and antimicrobial properties were evaluated and compared with that of the chemically synthesized Ag-NPs (Figure 1). The as-synthesized Ag-NPs were characterized using various microscopic and spectroscopic techniques. Furthermore, their microbicidal properties were tested against *Micrococcus luteus*, *Staphylococcus aureus*, *Escherichia coli*, and *Pseudomonas aeruginosa*.

## 2. Results and Discussion

### 2.1. UV-Vis Spectral Analysis

**Figure 2:**
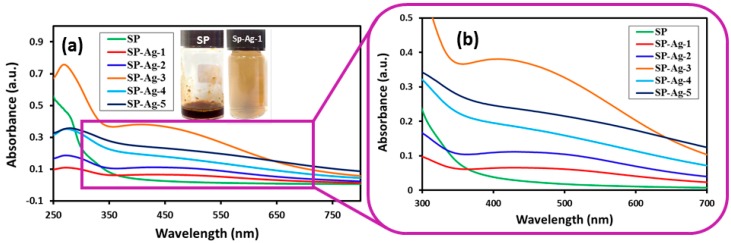
(**a**) UV-Vis spectra of pure *S. persica* L. RE (SP, green line). SP-Ag-1 (red line), SP-Ag-2 (blue line), SP-Ag-3 (orange line), SP-Ag-4 (cyan line), SP-Ag-5 (dark blue line), are the UV spectra of pure Ag-NPs prepared with 0.1 mL, 0.5 mL, 1.0 mL, 2.0 mL, and 3.0 mL RE, respectively; (**b**) Magnified image of Figure 2a. All the reactions were carried out for a period of 2 h, and the samples for the UV measurements were taken after final workup.

**Figure 3:**
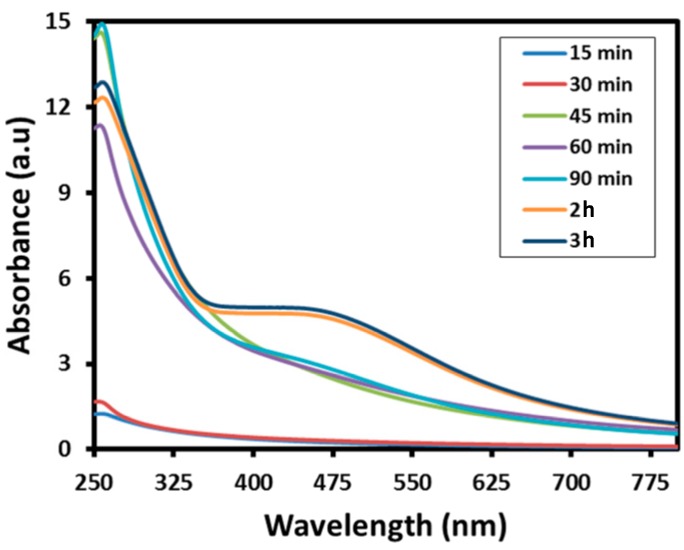
Kinetic reaction study of the (UV-Vis) absorption spectra of the silver nanoparticles (SP-Ag-3) during the synthesis at various time intervals.

The environmentally friendly preparation of Ag-NPs was carried under reflux conditions using the *S. persica* L. RE. The reduction of Ag ions was confirmed by the obvious change in the color of the reaction mixture from light brown to dark brown. Notably, no color change was observed in the absence of RE, under a similar set of conditions. Initially, the formation of Ag-NPs was further confirmed by UV analysis, which is a significant method to observe the development and stability of Ag-NPs. For this purpose, the absorption spectra of pure RE and various samples of green synthesized Ag-NPs (SP-Ag) prepared by using different volumes of RE was measured as shown in Figure 2. The UV analyses of these samples have revealed that, in the beginning of the reaction, the process of nucleation started swiftly, which resulted in the fast formation of Ag-NPs for the first 60 min. This is clearly reflected in the UV spectra of Ag-NPs, where the intensities of the absorption bands at ~450 nm gradually increases during the first 60 min of the reaction (Figure 3). After two hours, the process of nucleation was slowed down and no further change in the intensity of the absorption band was observed even after several hours, which clearly indicated the completion of reaction.

To evaluate the effect of the volume of the RE on the preparation of Ag-NPs, various samples of Ag-NPs were prepared by using different volumes of *S. persica* L. RE. These samples of SP-Ag were synthesized by using 0.1 mL (SP-Ag-1), 0.5 mL (SP-Ag-2), 1.0 mL (SP-Ag-3), 2.0 mL (SP-Ag-4), and 3.0 mL (SP-Ag-5) of miswak RE taken from the stock solution as mentioned in the experimental section. Notably, the amount of AgNO_3_ was kept constant in all these experiments. Initial UV analyses of these samples have clearly demonstrated that the formation of Ag-NPs only occurs when a lower volume of RE—between 0.1 and 3 mL (Figure 2)—is used. Whereas, at higher volumes of RE up to 5 mL, the formation of NPs will cease to occur, which was also confirmed by UV analysis (data is not shown). Furthermore, the UV analysis also shed some light on the size of NPs. 

For instance, it is reported in the literature that an increase in the particle size is usually associated with a broad peak at a higher wavelength, whereas a narrow line at a shorter wavelength represents smaller particle size [21]. In order to study this effect, we varied the volume of RE from 0.1 to 3 mL. In the case of Ag-NPs prepared from *P. glutinosa* PE [21], it has been observed that the broadening of the peak decreases with increasing the volume of PE. However, we did not observe a similar effect in the case of Ag-NPs prepared from *S. persica* L. RE. Contrarily, in this case, when the volume of RE was increased, the absorption band becomes broader and also shifted to higher wavelengths, which points towards the deteriorating size of Ag-NPs. Additionally, upon further increasing the volume of RE above 3 mL, the reaction did not occur.

These results clearly indicate that *S. persica* L. RE has opposite effects when compared to the *P. glutinosa* PE. This is probably due to the presence of the additional inorganic residual moieties (details of which is given below) in the *S. persica* L. RE, which are not present in *P. glutinosa* PE [21]. When the volume of the RE is increased during the synthesis of Ag-NPs, the amount of these inorganic moieties also increases, which possibly inhibits the formation of Ag-NPs. These results clearly suggest that different types of PEs may have dissimilar effects on the size and morphology of NPs due to the presence of diverse phytoconstituents. Therefore, the identification of active constituents responsible for the formation of NPs may greatly facilitate the controlled synthesis of NPs using PE.

### 2.2. FT-IR Analysis

**Figure 4:**
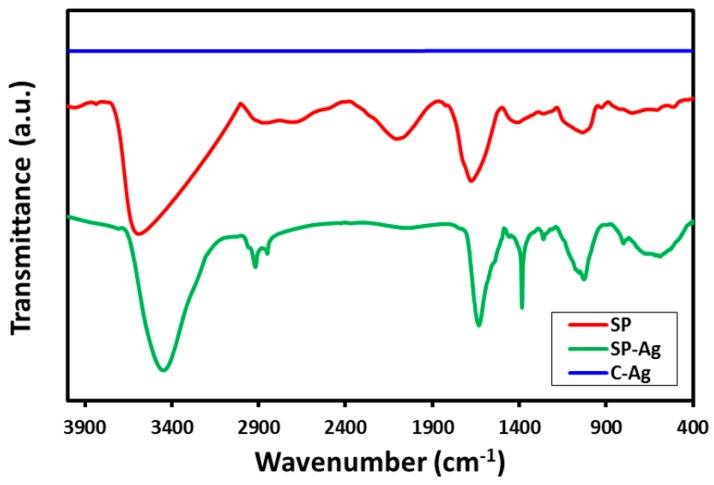
Comparison of the IR spectra of pure *S. persica* L. RE (SP, red line), Green synthesized Ag-NPs (SP-Ag, green line), and chemically synthesized Ag-NPs (C-Ag, blue line).

The twin role of the RE as a green reductant and stabilizing agent was established by comparing the Fourier transformed infrared (FT-IR) spectra of pure *S. persica* L. RE, green synthesized Ag-NPs (SP-Ag-NPs), and chemically synthesized Ag-NPs (C-Ag) as shown in Figure 4. In order to exclude any possibility of the presence of residual unbound phytoconstituents on the surface of the green synthesized Ag-NPs, the purified SP-Ag-NPs were redispersed in DI (deionized) water via sonication for 15 min. Subsequently, the NPs were isolated via centrifugation for 30 min at a speed of 9000 rpm. A similar process was repeated three times to isolate pure Ag-NPs.

As shown in Figure 4, the FT-IR spectrum of green synthesized Ag-NPs (SP-Ag) is remarkably similar to the FT-IR spectrum of pure *S. persica* L. RE, except slight minimal shifts in a few peaks.This striking resemblance between these two spectra clearly suggests that some of the residual phytomolecules of the *S. persica* L. RE remained attached on the surface of the green synthesized Ag-NPs. This is further confirmed by comparing the FT-IR spectrum of SP-Ag with that of chemically synthesized Ag-NPs (C-Ag). Since, the chemical synthesis was carried out without using any organic moiety as a stabilizing agent; no functional groups were bound to the surface of C-Ag. Therefore, the FT-IR spectrum of C-Ag does not possess any signal. The FT-IR spectrum of RE exhibit several absorption peaks at different locations including at, 3627 cm^−1^ (hydrogen bound OH), ~2933 cm^−1^ (C–H, asymmetrical stretch), ~2132 cm^−1^ (C≡C stretch) ~1698 cm^−1^ (C=O stretch) 1398 cm^−1^ (C–O stretch) ~1000 cm^−1^ (C–O) which correspond to various oxygen containing functional groups. Majority of these peaks are also present in the IR spectrum of SP-Ag with some minimal shifts. For instance, all the aforementioned peaks are slightly shifted in the IR spectrum of SP-Ag and appeared at ~3504 cm^−1^, ~2959 cm^−1^, ~1663 cm^−1^, ~1074 cm^−1^. Therefore, the presence of these peaks in the IR spectrum of SP-Ag clearly points towards the successful dual role of the *S. persica* L. RE, both as a reducing and capping agent.

### 2.3. Powder X-ray Diffraction

**Figure 5:**
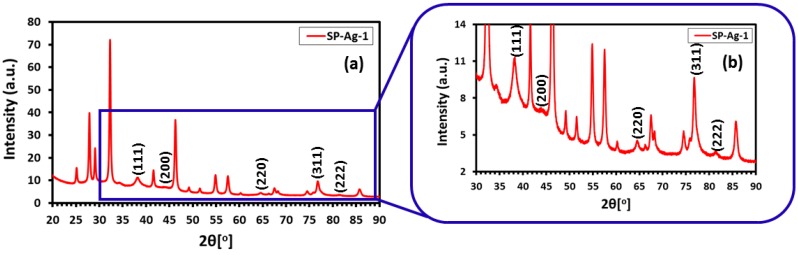
(**a**) X-ray diffraction (XRD) diffractogram of SP-Ag-NPs; (**b**) Magnified image of Figure 5a.

Additionally, the SP-Ag was also characterized by X-ray diffraction (XRD) analysis. The diffractogram of SP-Ag exhibited several intense diffractions (Figure 5), which not only confirms the crystallinity of the sample but also established the identity of the NPs. Particularly, the five discrete reflections at 37.50° (111), 44.13° (200), 64.30° (220), 76.89° (311), and 81.2° (222), clearly confirms the formation of the face-centered cubic (fcc) structure of the Ag-NPs. Notably, the growth direction of the nanocrystals is represented by the most intense reflection at 37.50° (111) [40]. Similar reflections also found in the XRD spectrum of chemically synthesized Ag-NPs (see Section 2.5), which confirms the identity of NPs synthesized from this method. However, in the XRD spectrum of green synthesized Ag-NPs additional reflections are also found apart from the reflections belonging to Ag-NPs (Figure 5). The presence of these additional reflections is attributed to residual moieties of the *S. persica* L. RE.

### 2.4. TEM and EDX Analysis

**Figure 6:**
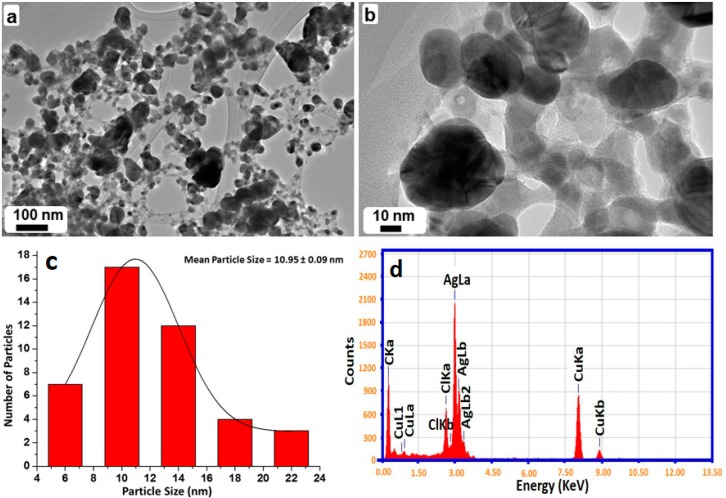
Transmission electron microscopy (TEM) and HRTEM (high-resolution) images of the Ag-NPs (**a**) overview; (**b**,**c**) Particle size distribution graph of Ag nanoparticles; (**d**) energy-dispersive X-ray spectroscopy (EDX) of Ag-NPs.

The size and morphology of SP-Ag-NPs prepared with 1 mL of RE (SP-Ag-3) were evaluated by transmission electron microscopy (TEM). Figure 6a shows an overview of the green synthesized Ag-NPs, whereas, Figure 6b, c shows the spherical morphology of the NPs, within a range of 10–20 nm. Mostly, the NPs are well distributed however; few of them were found to be agglomerated. It was revealed that the agglomeration of NPs decreases with an increase in the RE volume, which was also ascertained by UV analysis. Furthermore, the elemental composition of the green synthesized sample was also determined by energy-dispersive X-ray spectroscopy (EDX), which reveals the clear elemental composition profile of the SP-Ag-NPs as shown in Figure 5d. The intense signal at 3 keV strongly suggests that Ag was the major element, which has an optical absorption in this range due to the surface plasmon resonance (SPR) [21]. It was also noted that other signals were also found in the range 0.0–0.5 keV, which represent the typical absorption of oxygen and carbon, thus signifying the presence of the residual phytoconstituents of *S. persica* L. RE (as a capping ligand) on the surfaces of the NPs.

### 2.5. XRD, SEM, and EDX Analysis of S. persica *L.* Root Extract

**Figure 7:**
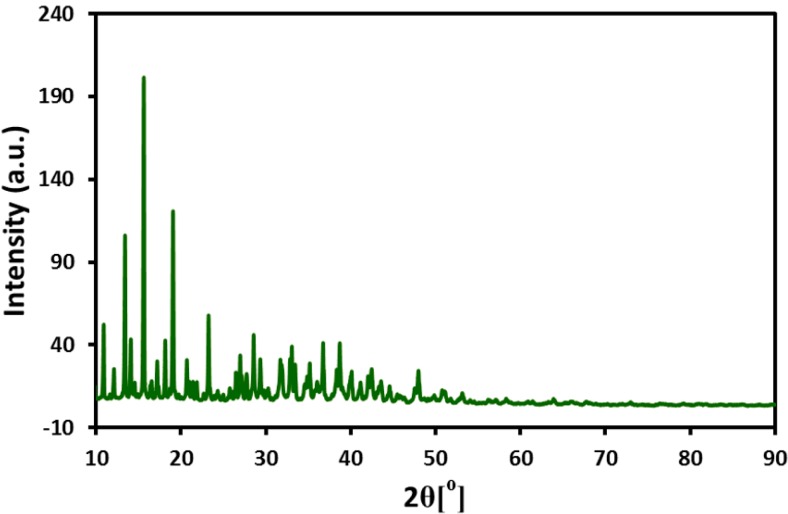
XRD diffractogram of the white colored inorganic residue obtained from the *S. persica* L. RE.

**Figure 8:**
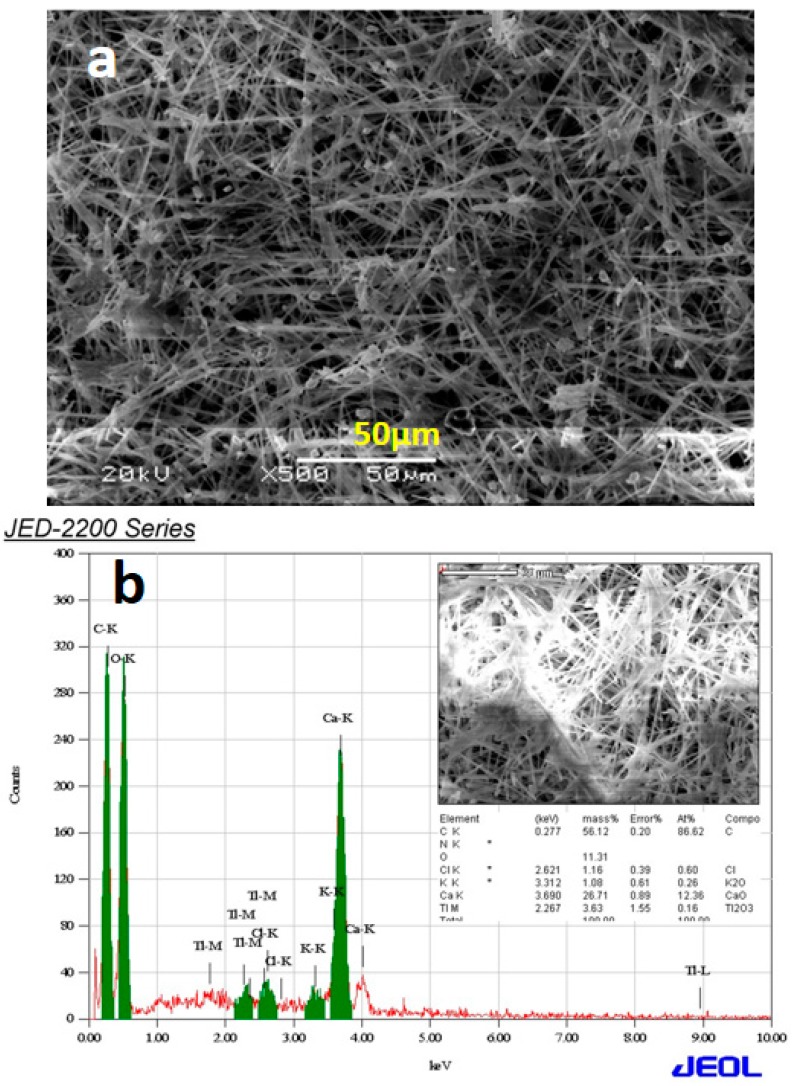
(**a**) Scanning electron microscopy (SEM) image; and (**b**) EDX of *S. persica* L. root extract.

During the extraction process *S. persica* L. RE, before evaporation of solvent (water) from the RE solution, a white powder precipitated out in large amount. The powder is separated out from the solution, which is subsequently evaporated to get the pure RE for the synthesis of NPs. The powder is subjected to various characterization techniques including XRD (Figure 7), scanning electron microscopy (SEM), and EDX (Figure 8), however its complete identity is still unknown. The results obtained so far indicate that, this could be calcium based mineral (Figure 8). However, in order to completely confirm the identity of this compound, it needs to be subjected to thorough investigation, which is already ongoing.

The additional reflections in XRD spectrum of Ag-NPs synthesized by *S. persica* L. RE may be due to the presence of the aforementioned inorganic residual moieties, which is suspected to be calcium based mineral (further studies are being conducted to ascertain the identity of this compound). The residual organic moieties which were also present on the surface of the Ag-NPs synthesized from *P. glutinosa* PE [21] did not contribute any additional reflections in the respective XRD spectrum. Although extra care was taken to remove this compound from the pure RE, however, some residual amount of this particular compound might still be present in the RE used for the synthesis of Ag-NPs. During the synthesis of Ag-NPs, the residual compound may be bound to the surface of the NPs and thus contributes to the additional reflection in the XRD spectrum of Ag-NPs synthesized by *S. persica* L. RE. This was further confirmed by comparing the XRD spectra of precipitate obtained from the aqueous solution of RE and the Ag-NPs obtained by using RE. The majority of the additional reflections present in the XRD spectrum of Ag-NPs were also present in the spectrum of precipitated powder from RE aqueous solution. This clearly suggests that the residual amount of precipitated powder not only remained in the pure extract but also bound to the surface of the metallic NPs.

### 2.6. UV-Vis and XRD Analysis of Chemically Synthesized Ag-NPs

**Figure 9:**
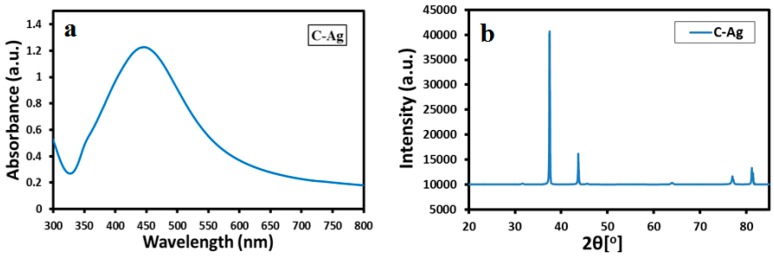
(**a**) UV-Vis absorption spectra; and (**b**) XRD diffractogram of chemically synthesized silver nanoparticles.

**Figure 10:**
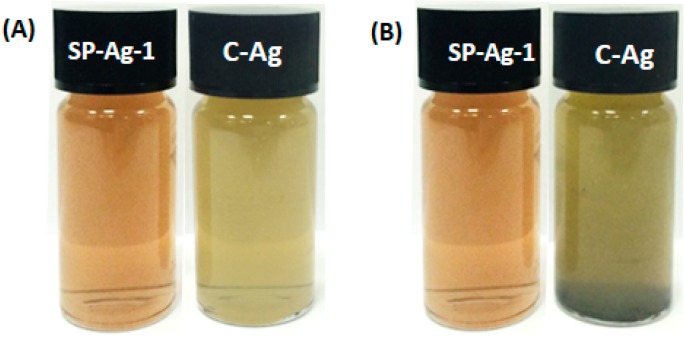
The green synthesized Ag-NPs display better dispersion quality when compared to the chemically synthesized Ag-NPs (**A**) 0 min; (**B**) After two days.

Furthermore, the *S. persica* L. RE mediated synthesized Ag-NPs are also compared with chemically synthesized Ag-NPs (Figure 9), which were obtained by the reduction of AgNO_3_ using sodium citrate. The formation of chemically synthesized Ag-NPs is confirmed by both UV-Vis and XRD analysis as shown in Figure 9. The UV-Vis absorption spectrum of chemically obtained Ag-NPs displayed a relatively sharp absorption band around 420 nm, unlike the green synthesized Ag-NPs (SP-Ag-3), which exhibits the absorption at ~450 nm. The occurrence of sharp absorption band at lower wavelength in the case of chemically synthesized Ag-NPs clearly points towards the smaller size of the NPs when compared to the green synthesized Ag-NPs. Furthermore, the XRD spectrum of C-Ag exhibits prominent reflection of Ag-NPs, which indicates the formation of face-centered cubic (*fcc*) structure of the Ag-NPs. Hence, the presence of similar reflections in the XRD spectrum of green synthesized Ag-NPs (Figure 5), apart from several additional reflections confirms that identical phases of Ag-NPs were obtained by both chemical and green syntheses. Although, the C-Ag-NPs exhibit smaller sizes and identical phases, however, the green synthesized Ag-NPs displayed better dispersion quality when compared to the chemically synthesized Ag-NPs, which is an important parameter in the enhancement of the biological activity of the Ag-NPs (Figure 10).

To compare the dispersion quality of both green synthesized Ag-NPs and chemically synthesized Ag-NPs, freshly produced Ag-NPs from both methods (Figure 10A) were dispersed in DI water. The samples for the comparison study were prepared by dispersing 5 mg of each substance in 10 mL of DI water via sonication. High quality dispersion was achieved for the green synthesized Ag-NPs as compared to the chemically synthesized Ag-NPs. After two days, the chemically synthesized Ag-NPs became unstable, whereas the green synthesized Ag-NPs still exhibited excellent dispersibility (Figure 10B). The enhanced dispersion quality of green synthesized Ag-NPs is attributed to the presence of residual organic moieties of the RE, which act as functionalization ligands and stabilize the as-prepared NPs in aqueous solution.

### 2.7. Microbicidal Activity of Silver Nanoparticles

**Figure 11:**
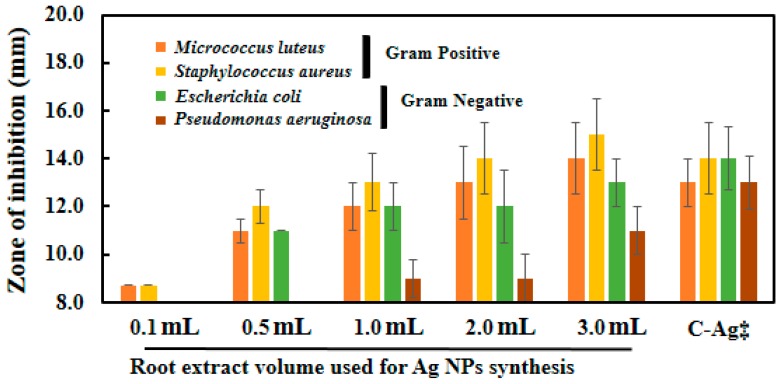
Antimicrobial activity of Ag-NPs against Gram-positive and Gram-negative bacteria as determined by Kirby-Bauer assay. Antimicrobial activity of the SP-Ag-NPs (100 µg/well) is also compared with C-Ag-NPs‡. The *S. persica* L. RE do not show any antimicrobial activity at the highest concentration used for the synthesis of Ag-NPs (i.e., 300 µg/mL).

Ag-NPs synthesized with different volume of *S. persica* L. RE exhibited good antimicrobial activity against all the organisms tested. The diameters of zone of inhibitions produced by Ag-NPs synthesized with different volumes of RE against both Gram-negative (*E. coli* and *P. aeruginosa*) and Gram-positive (*M. luteus* and *S. aureus*) bacterial strains is shown in Figure 11. It is evident from the data presented in Table 1, that RE does not exhibit any antimicrobial activity at the highest volume tested containing 300 µg of RE used for the green synthesis of the Ag-NPs. Whereas, the antimicrobial activities of the green synthesized Ag-NPs increases with the increasing volume of the RE. Notably, the highest activity was reported with the Ag-NPs synthesized using 3 mL of RE. Furthermore, the microbicidal potential of the chemically prepared Ag-NPs was also compared with that of the green synthesized Ag-NPs. The chemically synthesized Ag-NPs exhibited comparable, or slightly lower, antibacterial activity than the green synthesized Ag-NPs (Ag-NPs synthesized with 3 mL of RE). The results obtained in this study shows that, a higher antibacterial activity of the green synthesized Ag-NPs was observed against the tested Gram-positive bacteria, compared to the tested Gram-negative bacteria.

Increasing antimicrobial activity of Ag-NPs with the increasing volumes of RE used to synthesize the NPs was also observed in our previous study on *Pulicaria glutinosa* [41], mainly due to the increased solubility of the NPs. Although, in this study, the root extract of *S. persica* L., a plant with known antimicrobial activity and traditionally used for oral hygiene, was used [42]. However, the role of the aqueous extract was largely as a reductant for the synthesis of NPs as the concentration used for the synthesis was too low to show its own antimicrobial activity (10–300 µg/mL). It has been demonstrated in an earlier study that the aqueous RE of *S. persica* L. exhibited considerable antimicrobial activity at concentrations range of 50–400 mg/mL [43]. In this study, antimicrobial activity of the RE was tested at a very low concentration (300 µg/mL, which is ~100 times lower), therefore, no antimicrobial activity was observed. Hence, it can be concluded here that the increased activity of the Ag-NPs was mainly due to the improved solubility of the Ag-NPs rather than the microbicidal potential of the RE itself used for the synthesis of NPs. Higher antimicrobial activity against Gram-positive bacteria tested was observed than the Gram-negative bacteria. This may be due to the production of lipopolysaccharide (LPS) by Gram-negative bacteria, which may absorb the NPs on the outer surface of the bacterial cells. We obtained similar results in our previous studies on CuO and CoO nanostructures [35,44].

## 3. Experimental Section

### 3.1. Materials

The *S. persica* L. roots, which are widely available in the southern zone of Saudi Arabia, were procured from a local herbal market at Batha, Riyadh, Saudi Arabia. The identity of the plant was confirmed by an expert plant biologist and the details were reserved in our research laboratory with the sample coupon number KSUHZK-302. AgNO_3_ (99.8%, Sigma Aldrich, St. Louis, MO, USA), tri-sodium citrate and other solvents used in this work were purchased from Sigma Aldrich and were used as purchased.

### 3.2. Methods

#### 3.2.1. Preparation of *S. persica* L. RE

To begin with, freshly obtained *S. persica* L. roots were chopped into small pieces. Subsequently, the chopped pieces (1.3 kg) were drenched in DI water (3 L) and refluxed for ~4–5 h. After reflux the resultant solution was sieved and dried at 50 °C under reduced pressure in a rotary vacuum evaporator. Finally, a dark brownish gluey extract (30.0 g) was obtained, which was stored at 0–4 °C for further use. In order to use this extract for the preparation of Ag-NPs, a stock solution was prepared by dissolving 1.0 g of as-obtained RE in 10 mL of DI water.

#### 3.2.2. Green Synthesis of Silver Nanoparticles

The silver nanoparticles were synthesized by the addition of 1 mL of *S. persica* L. RE (from the stock solution) to 49 mL aqueous solution containing 84.935 mg (0.5 mM) of AgNO_3_ in a 150 mL flask. The resultant round-bottom flask was equipped with a magnetic stir bar and mounted on a cooling condenser. The mixture was stirred for ~2 h at 90 °C. While stirring, the color of the reaction mixture changed instantly from pale yellow to dim brown and thereafter no color change was witnessed until the end of the reaction (even after two hours). Subsequently, the reaction mixture was allowed to cool down and subjected to centrifugation at 9000 rpm (Hermle 236HK, Wehingen, Germany) at room temperature. Thereafter, the acquired product was washed several times (three times) with DI water. Finally, a black powder was achieved, which was dried overnight at 80 °C in an oven. In order to understand the effect of the volume of RE on the preparation of Ag-NPs, several experiments were carried out by taking different amounts of RE. For instance, various experiments were carried out by mixing 0.1, 0.5, 1.0, 2.0, and 3.0 mL of RE (from the stock solution) and 0.5 mmol of AgNO_3_ (84.935 mg) in particular amount of DI water to make up a total volume of 50 mL. All experiments were executed at 90 °C.

#### 3.2.3. Chemical Synthesis of Silver NPs

In order to compare the dispersion quality and the biological activity of both green synthesized and chemically synthesized Ag-NPs. A fresh sample of Ag-NPs was prepared by using tri-sodium citrate as reducing agent. In this method, spherical Ag-NPs were prepared according to the literature [45]. For instance, the reduction of AgNO_3_ was carried out by using tri-sodium citrate as reducing agent at boiling temperature. To begin with, 50 mL aqueous solution of AgNO_3_ with 0.001 M was prepared in a 100 mL round bottom flask. The reaction flask was mounted with a cooling condenser and the solution was allowed to reflux at boiling temperature (100 °C). To this boiling solution, 5 mL of 1% trisodium citrate was added slowly. The resultant reaction mixture was kept under constant stirring at 100 °C. Finally, the reaction was stopped when the solution changed to a greenish yellow color. The resultant solution was allowed to cool down and the product was isolated via centrifugation. The Ag-NPs obtained by this method are referred to as C-Ag throughout this article.

### 3.3. Methods of Characterization

#### 3.3.1. UV-Vis Spectroscopy

Optical measurements were carried out by using UV-visible spectrophotometer (Perkin Elmer lambda 35, Waltham, MA, USA). The measurements were carried out in quartz cuvettes by using double distilled water as a reference solvent. In order to perform the kinetic studies, the UV samples were prepared by dispersing 1.0 mL of crude mixture (the mixture collected during the reaction or at the end of the reaction) in 9.0 mL of DI water via sonication for 15 min. Furthermore, UV analysis of pure Ag-NPs was also carried out to confirm their identity. For this purpose, the stock solution of as-prepared Ag-NPs was prepared by dispersing 5.0 mg of pure Ag-NPs in 5.0 mL of distilled water via 1 h sonication. Finally, the sample for the UV analysis of pure Ag-NPs was prepared by diluting 2 mL of stock solution in 8 mL of DI water.

#### 3.3.2. Transmission Electron Microscopy (TEM)

To identify the size and morphology of the as-prepared Ag-NPs, TEM analysis was carried out by using JEM 1101 transmission electron microscope (JEOL, Tokyo, Japan). The specimen for the TEM analysis was prepared by dissolving 5 mg of Ag-NPs in 10 mL of ethanol. In order to prepare the grid for TEM measurement, a drop of as-prepared solution is placed on a grid (copper), which was then dried out at 80 °C for 6.0 h in an oven.

#### 3.3.3. X-ray Diffraction (XRD)

XRD diffractograms were obtained by using an Ultima IV X-ray powder diffractometer (Rigaku, Tokyo, Japan). During these measurements Cu Kα (λ = 1.5418 Å) was used as radiation source.

#### 3.3.4. Fourier Transform Infrared Spectrometer (FT-IR)

A Perkin Elmer 1000 FT-IR instrument was used to identify the phytomolecules present in *S. persica* L. root extract. To measure the FT-IR spectra of pure Ag-NPs, the as-prepared Ag-NPs were frequently washed with DI water, in order to remove any unbound extract or free biomass residue from the surfaces of NPs. The product obtained was centrifuged at 9000 rpm for 30 min and dried out. In order to prepare a pellet for the FT-IR analysis, the purified Ag-NPs were mixed with KBr. Finally, the background correction of the collected IR spectra was performed by using blank KBr pellet as a reference.

### 3.4. Microbicial Activity

Antibacterial activity of the engineered nanoparticles was determined by modified Kirby-Bauer disk diffusion method. Briefly, cultures of two Gram-negative bacteria namely *E. coli* ATCC 25922, *Ps. aeruginosa* ATCC 75853, and two Gram-positive bacteria—namely, *M. luteus* ATCC 10240 and *S. aureus* ATCC 92213—were grown in sterile Luria broth, nutrient broth, Müller-Hinton broth, and nutrient broth, respectively. *E. coli* and *Ps. aeruginosa* were grown on a rotary shaker at 150 rpm at 37 °C while *M. luteus* and *S. aureus* were grown at 30 °C. Agar plates were seeded with the mixture of exponentially growing test organisms (10^5^ colony-forming units/mL) with soft agar (0.7% agar). Plates were left standing for 10–15 min at room temperature for the agar to solidify. Wells 8 mm in diameter were punched into the soft agar layer for testing the antimicrobial activity of nanomaterial. Using a micropipette, 100 μg of the Ag-NPs synthesized with different volumes of RE were added to each well on the plates. Water was used as a negative control while 300 μg of the root extract and 100 μg of the chemically synthesized silver nanoparticles were also included in the experiment for meaningful comparisons. Plates were incubated overnight at their respective temperatures for one to two days and zones of inhibition were measured using a ruler. All the experiments were performed in triplicate and the values reported are the mean values of three independent experiments.

## 4. Conclusions

In this study, an environmentally friendly method to the preparation of Ag-NPs using *S. persica* L. RE has been described. With this method, high quality, spherical Ag-NPs were obtained under facile conditions without applying any hazardous chemicals as reductants or stabilizing agents. The NPs were prepared using a very low volume of RE, exhibiting lower size and less aggregation, whereas, the reduction of AgNO_3_ was stopped at the higher volume of the RE. Furthermore, the study of antimicrobial activities of both chemically and green synthesized Ag-NPs, as well as the pure RE, against various microorganisms has revealed that the *S. persica* L. RE does not possesses antibacterial activity of its own at lower concentrations. However, it exerts significant effects on the antimicrobial property of the green synthesized Ag-NPs. In addition, the green synthesized Ag-NPs exhibited slightly higher or equivalent antimicrobial activities in comparison to the antimicrobial activities of the chemically synthesized Ag-NPs. Notably, by virtue of its dual nature, *S. persica* L. RE not only controls the size but also facilitates the stabilization of the Ag-NPs. This significantly enhances the penetration ability of the NPs into the bacterial cell wall and thus results in the improved microbicidal activity of RE capped Ag-NPs. Therefore, due to the high oxidizing ability, low cost, and abundancy, *S. persica* L. RE has great potential for the reduction of various other metal salts and can be possibly applied for the synthesis of various metallic NPs and other functional nanomaterials.

## Figures and Tables

**Table 1:** Microbicidal activity of Ag-NPs synthesized with various volumes of root extract.

RE* Volumes used for Ag-NPs Synthesis	Zone of Inhibition (mm) ^a^			
	Gram Positive		Gram Negative	
	*M. luteus*	*S. aureus*	*E. coli*	*P. aeruginosa*
RE ^b^	8	8	8	8
0.1 mL	8.7	8.7	8	8
0.5 mL	11	12	11	8
1.0 mL	12	13	12	9
2.0 mL	13	14	12	9
3.0 mL	14	15	13	11
C-Ag ^c^	13	14	14	13

^a^ The initial size of the wells is 8 mm; ^b^ RE denotes root extract; ^c^ Denotes chemically synthesized Ag-NPs.
